# Predictive Value of HbA1c and Metabolic Syndrome for Renal Outcome in Non-Diabetic CKD Stage 1–4 Patients

**DOI:** 10.3390/biomedicines10081858

**Published:** 2022-08-02

**Authors:** Chi-Chih Hung, Yen-Yi Zhen, Sheng-Wen Niu, Kun-Der Lin, Hugo You-Hsien Lin, Jia-Jung Lee, Jer-Ming Chang, I-Ching Kuo

**Affiliations:** 1 Division of Nephrology, Department of Internal Medicine, Kaohsiung Medical University Hospital, Kaohsiung Medical University, Kaohsiung 807, Taiwan; chichi@kmu.edu.tw (C.-C.H.); a0928306234@gmail.com (Y.-Y.Z.); 950138kmuh@gmail.com (S.-W.N.); yukenlin@yahoo.com.tw (H.Y.-H.L.); jjlee@kmu.edu.tw (J.-J.L.); jemich@kmu.edu.tw (J.-M.C.); 2 Department of Internal Medicine, Kaohsiung Municipal Ta-Tung Hospital, Kaohsiung Medical University, Kaohsiung 801, Taiwan; 3 Division of Endocrinology and Metabolism, Department of Internal Medicine, Kaohsiung Medical University Hospital, Kaohsiung Medical University, Kaohsiung 807, Taiwan; berg.kmu@gmail.com

**Keywords:** HbA1c, metabolic syndrome, chronic kidney disease

## Abstract

Glycated hemoglobin (HbA1c) levels are commonly used to indicate long-term glycemic control. An HbA1c level of 6.5–5.7% is defined as pre-diabetes and is proposed as a criterion for diagnosing metabolic syndrome (MetS). However, HbA1c levels can be affected by chronic kidney disease (CKD). Whether HbA1c is associated with clinical outcomes in nondiabetic CKD patients with or without MetS is still unknown. This study included 1270 nondiabetic CKD stage 1–4 Asian patients, divided by HbA1c and MetS. Through linear regression, HbA1c was positively associated with age, waist circumference, hemoglobin levels, and C-reactive protein and was negatively associated with malnutrition–inflammation. HbA1c levels were 5.5% (0.6%) and 5.7% (0.6%) in non-MetS and MetS, respectively (*p* < 0.001). In Cox regression, higher-level HbA1c was associated with worse composite renal outcome in MetS patients, but with better renal outcome in non-MetS patients: Hazard ratio (HR) (95% confidence interval [CI]) of HbA1c ≥5.7%, compared with HbA1c <5%, was 2.00 (1.06–3.78) in MetS and 0.25 (0.14–0.45) in non-MetS. An association between HbA1c and all-cause mortality was not found. In conclusion, higher HbA1c levels are associated with worse renal outcomes in nondiabetic CKD stage 1–4 patients modified by the presence of MetS.

## 1. Introduction

Glycated hemoglobin (HbA1c) is a reflection of long-term glucose maintenance, which is affected primarily by postprandial glucose excursion especially in nondiabetic individuals [1]. According to guidelines from the American Diabetes Association (ADA), an HbA1c level below 5.7% is considered normal, whereas levels between 5.7% and 6.4% are considered prediabetes. Individuals with prediabetes identified by elevated HbA1c, but not fasting plasma glucose, have been reported with cardiovascular disease and mortality as endpoints [2,3]. Additionally, HbA1c within the nondiabetic range has been known to constitute a risk factor for incidents of chronic kidney disease (CKD), cardiovascular disease (CVD) risk, and all-cause mortality in several cohort- or community-based studies [4,5,6,7,8,9,10]. Another nondiabetic cohort, with mostly CKD stages 3–4, showed that patients with HbA1c >5.7% have a higher risk of mortality than those with lower values [11]. Altogether, it is hypothesized that increased glucose excursions of nondiabetic hyperglycemia rather than stably elevated glucose levels are directly related to these complications.

Elevated fasting glucose is a component of metabolic syndrome (MetS), which refers to metabolic disturbances with the distinct feature of insulin resistance, and may indicate a predisposition toward central obesity [12]. MetS contributes to cascade reactions including lipotoxicity, oxidative stress, chronic inflammation, apoptosis, and endothelial dysfunction, which may consequently accelerate atherosclerosis- and glomerulosclerosis-related kidney damage [13,14]. MetS itself, independent of individual components, is also found to be a powerful predictor of CKD [15,16,17]. Moreover, in a previous meta-analysis, the presence of MetS was associated with the future development of incident CKD, and the risk estimate was remarkably increased as the number of components of MetS increased [18]. These clinical findings may indicate that the components of MetS work synergistically to increase the risk of renal damage.

Along with this background, accumulating evidence has shown the clinical usefulness of HbA1c measurement in predicting cardiometabolic risks in nondiabetic populations. Additionally, some studies have proposed potential diagnostic criteria for MetS through analysis of HbA1c levels [19,20]. In clinical practice, HbA1c levels are easily accessible and are not generally influenced by daily fluctuations. Nevertheless, there are still limited data about the predictive ability of HbA1c for long-term risks of clinical outcomes in nondiabetic CKD patients. Our studies have demonstrated that the predictive ability of HbA1c in diabetic CKD was influenced by advanced CKD stage and anemia [21]. Therefore, this study aimed to evaluate the predictive value of HbA1c measurement in nondiabetic CKD stage 1–4 patients with or without MetS.

## 2. Materials and Methods

### 2.1. Participants and Measurements

This is a prospective observational study that analyzed data from enrolled patients from two affiliated hospitals of Kaohsiung Medical University in southern Taiwan. These patients were participating in the Integrated CKD Care Program Kaohsiung for Delaying Dialysis. The study was conducted from 11 November 2002 to 31 May 2009, with follow-ups occurring until 31 December 2014, as previously described [22]. We included patients with stage 1–5 CKD whose estimated glomerular filtration rate (eGFR) was calculated by the Modification of Diet in Renal Disease (MDRD) formula. Eligible patients were followed up for more than 3 months to confirm the presence of CKD. Patients were excluded if they had acute kidney injury, defined as a decrease of >50% in the eGFR within 3 months, or if they had undergone renal replacement therapy (RRT) before their first visit. There were 3659 CKD stage 1–5 patients in this original cohort. However, based on this study’s hypothesis, we excluded patients diagnosed with diabetes or who had been treated with antidiabetic medications. Accordingly, 1270 nondiabetic CKD stage 1–4 patients were analyzed. The Institutional Review Board of the Kaohsiung Medical University Hospital approved the study protocol, and informed consent was obtained from all of the patients participating in the study.

The baseline demographic features, relevant comorbidities, blood pressure (BP), and waist circumference (WC) were collected by trained nurses in the clinic at the time of patient enrollment. The presence of cardiovascular diseases (CVD) was defined as having a clinical diagnosis of heart failure, acute or chronic ischemic heart disease, or cerebrovascular disease. Hyperuricemia was defined as a plasma uric acid concentration of ≥8.0 mg/dL. The diagnosis of MetS was based on the modified National Cholesterol Education Program’s Adult Treatment Panel III (NCEP-ATP III) and was defined as requiring the presence of at least three of the following five criteria: (1) waist circumference of ≥90 cm for men and ≥85 cm for women; (2) triglyceride (TG) levels of ≥150 mg/dL; (3) high-density lipoprotein cholesterol (HDL-C) levels of <40 mg/dL for men and <50 mg/dL for women; (4) systolic BP of ≥130 mmHg, or diastolic BP of ≥ 85mmHg or treatment for previously diagnosed hypertension; and (5) fasting plasma glucose levels of ≥100 mg/dL. The malnutrition–inflammation score (MIS) is composed of 10 components included in four sections: nutritional history, physical examination, body mass index (BMI), and laboratory values (including serum albumin and total iron binding capacity) [23]. This study used MIS of ≥6 as the definition of moderate malnutrition–inflammation according to unpublished data linking to clinical outcomes in CKD, whereas the only other published paper on MIS in CKD suggested a cut-off at 8 [24]. In consideration of insulin resistance, several studies have reported that the triglyceride-glucose index (TyG) provided a reliable prediction of MetS and was even superior to homeostatic model assessment of insulin resistance (HOMA-IR) for identifying insulin resistance [25,26,27]. Therefore, we used the TyG index to reflect the status of insulin resistance, which was calculated by the equation: ln [fasting serum TG (mg/dL) × FPG (mg/dL)/2]. Other baseline biochemical covariates including hemoglobin (Hb), albumin, creatinine (Cr), fasting blood glucose, cholesterol, C-reactive protein (CRP), HbA1c, phosphorus, calcium, urine protein–creatinine ratio (UPCR), and uric acid levels were all obtained after midnight fasting.

### 2.2. Outcomes

Patients were prospectively followed for a 50% decline in eGFR from the baseline visit to the follow-up visit, end-stage renal disease (ESRD), all-cause mortality, loss to follow-up, or end of follow-up. ESRD was defined as the initiation of hemodialysis, peritoneal dialysis, or renal transplantation. The initiation of renal replacement therapy was confirmed by catastrophic illness cards. We used the MDRD equation for estimating kidney function: eGFR mL/min/1.73 m^2^ = 186 × serum Cr −1.154 × age −0.203, and ×0.742 (if the patient was female), or ×1.212 (if the patient was Black). All patients periodically underwent serial blood examinations during follow-up periods to evaluate declines in kidney function. The survival status and cause of death were determined on the basis of death certificates and the National Death Index.

### 2.3. Statistical Analysis

The summarized statistical results of the baseline characteristics of patients were expressed as counts and percentages for the categorical data, and means with standard deviation and medians with interquartile ranges (IQR) were determined for continuous variables with approximately normal distributions. Logarithmic transformation of variables with a skewed distribution (TG, UPCR, and CRP) was applied in analyses. A multivariable linear regression analysis was utilized to evaluate the relationship between HbA1c and other variables. HRs and 95% CIs from Cox proportional hazard model were stratified by HbA1c and used to estimate relative risks for composite renal outcomes and all-cause mortality. The rate of kidney function decline per year was assessed using the slope of eGFR obtained from a generalized linear mixed model. Each outcome was allowed to occur only once per participant. Covariates considered for possible confounders were used for adjustment. These included age, sex, eGFR, log-UPCR, CVD, cancer, severe liver disease, smoker, hypertension, malnutrition–inflammation, Hb, albumin, log-CRP, phosphorus, BMI, WC, mean BP, HDL, and log-TG. A *p* value of <0.05 was considered to be statistically significant. Statistical analyses were conducted using Statistical Package for Social Sciences Version 20.0 for Windows (SPSS Inc., Chicago, IL, USA).

## 3. Results

### 3.1. Participant Characteristics

Table 1 summarizes the baseline clinical and biochemical characteristics of the 1270 participants based on the presence of MetS and HbA1c levels. The mean ages were 58.0 ± 17.3 and 62.8 ± 14.2 years, the mean eGFRs were 40.1 (26.5–56.2) and 35.1 (24.5–47.9) mL/min/1.73 m^2^, and the UPCRs were 451 (162–1171) and 608 (218–1368) mg/g in patients without MetS and with MetS, respectively (all *p* > 0.05 between two groups). Patients with MetS were older and had a higher prevalence of CVD, hyperuricemia, and HTN; however, other parameters listed in Table 1 showed no difference when compared with patients without MetS. Moreover, among patients with MetS, HbA1c ≥5.7% was also related to higher hemoglobin levels, higher albumin levels, and lower prevalence of malnutrition–inflammation. During the mean follow-up period of 8.2 years, 141 (22.5%) patients without MetS reached renal outcomes compared with 182 (28.3%) patients with MetS, and 78 (12.4%) without MetS died compared with 110 (17.1%) with MetS, respectively.

### 3.2. Components of MetS in Participants

Table 2 summarizes the components of MetS and the related parameters according to the presence of MetS and HbA1c level. All MetS components and parameters, by definition, were worse in patients with MetS. Total cholesterol levels were similar in both groups. In patients without MetS, an HbA1c of ≥5.7% was associated with higher WC and fasting blood sugar levels. In patients with MetS, an HbA1c of ≥5.7% was associated with higher WC, BMI, and fasting blood sugar levels.

### 3.3. Relationship between Clinical and Biochemical Variables and HbA1c

Table 3 shows the result of multivariable linear regression of relevant clinical and biochemical covariates with HbA1c as the dependent variable. HbA1c level had a significantly positive relationship with MetS components, including waist criteria (β = 0.114; *p* = 0.001) and blood sugar criteria (β = 0.158; *p* < 0.001), as well as BP criteria (β = 0.092; *p* = 0.050). Additionally, HbA1c was also positively correlated with hemoglobin (β = 0.023; *p* = 0.019) and log CRP (β = 0.040; *p* = 0.038) and negatively correlated with malnutrition–inflammation (β = −0.056; *p* = 0.043). As for the relationship between HbA1c and insulin resistance, HbA1c was found to be positively correlated with the TyG index (β = 0.165; *p* < 0.001). No interaction effect from the presence of MetS on the relationship between HbA1c and other variables was found. Supplemental Table S1 shows the results of multivariable logistic regression of relevant clinical and biochemical covariates with HbA1c.

### 3.4. HbA1c Level and Its Association with Clinical Outcomes with or without MetS

Multivariable Cox proportional hazard regression analysis was used to examine the association of HbA1c and MetS with clinical outcomes (Table 4). The presence of MetS increased the risk of composite renal outcomes, with an HR of 1.31 (95% CI, 1.01–1.69; *p* = 0.04) using the same hazard regression model as in Table 4, whereas the risk was insignificant for all-cause mortality (Supplemental Table S2). In the non-MetS group stratified by HbA1c, the fully adjusted HRs of the HbA1c level 5–5.7% group and the ≥5.7% group for composite renal outcomes were significantly decreased with 0.49 (95% CI, 0.32–0.77; *p* < 0.01) and 0.25 (95% CI, 0.14–0.45; *p* < 0.01), respectively. By contrast, in the MetS group, the fully adjusted HRs of HbA1c level 5–5.7% and ≥5.7% for composite renal outcome were significantly increased by 2.04 (95% CI, 1.11–3.74; *p* < 0.01) and 2.00 (95% CI, 1.06–3.78; *p* < 0.01), respectively. However, there were no associations between HbA1c level and all-cause mortality in both the MetS and non-MetS groups.

## 4. Discussion

In this study’s nondiabetic CKD patients with MetS, we demonstrated that HbA1c of >5.0% was significantly associated with worse renal outcomes independent of conventional CKD risk factors. By contrast, for those without MetS, high HbA1c levels were instead associated with better renal survival. However, there was no obvious excess risk associated with all-cause mortality and HbA1c, whether in patients with MetS or without MetS. Importantly, this is the first study to demonstrate that MetS might be an effect modifier for the prediction of HbA1c with clinical outcomes in a large nondiabetic CKD cohort.

Glucose metabolism and insulin resistance in CKD are complex factors and may be mediated by multiple parameters irrespective of having diabetes. Examples include (1) decreased beta-cell response to blood glucose since early stage of CKD, (2) decreased renal insulin clearance, and (3) increased hepatic gluconeogenesis [28]. All of these parameters could indicate a predisposition for metabolic acidosis, uremic toxin accumulation, chronic inflammation, vitamin D deficiency, and decreased adiponectin in CKD state [28]. Resistance to insulin might be amplified by any progression of CKD [29]. As for the prevalence of MetS in CKD, the Chronic Renal Insufficiency Cohort (CRIC Study) showed that 65% of 3939 total participants fulfilled the diagnostic criteria, whereas 35% of those with MetS had no diabetes [30]. Another study involving 200 CKD stage 4 and 5 patients demonstrated an overall prevalence of MetS of 30.5%, and it was even more common in dialysis therapy settings [31]. However, we found a high prevalence of MetS (50.5%) even in the absence of diabetes in this study, but it should be noted that participants with MetS were older than those without MetS, which corresponds to the observed increased prevalence of MetS worldwide in aging populations [32]. Conversely, the presence of MetS conferred a remarkable risk for developing incident CKD in both nondiabetic and diabetic patients [15,17,18,33,34]. Even among CKD patients, Yun et al. displayed a higher risk of composite renal outcomes including ESRD in CKD stage 1–5 patients with both obesity and metabolic abnormality [35]. More recently, Koh et al. demonstrated that resolved MetS components had a decreased risk of ESRD, especially among the more prominent effects seen in those with advanced renal dysfunction, using the Korean general population insurance database [36]. Our findings are consistent with prior studies showing that the presence of MetS confers an increased risk for kidney function progression in nondiabetic CKD. Collectively, all of these findings may strengthen the evidence for the role of modulating metabolic risk in prevention of kidney damage.

HbA1c and fasting glucose levels represent different aspects of the glycemic burden. HbA1c levels change slowly and reflect averages over 2–3 months and depict a chronic glycemic profile, whereas fasting glucose levels may be affected by some acute perturbations [37]. In the absence of a DM diagnosis, epidemiological reports from Korea detected better diagnostic accuracy of HbA1c for MetS [19,20,38], even within the category of patients with normal fasting glucose levels [20]. Particularly, it has also been established that elevated HbA1c levels predicted long-term risks of CKD, cardiovascular disease and death from any cause, even superior to fasting glucose levels in some circumstances [8,9,10]. Nevertheless, assessment of glycemia by HbA1c levels should consider the associated factors that can potentially bias the measure toward either low or high ranges. For example, it is generally recognized that CKD-associated abnormalities that can alter red blood cell (RBC) turnover, or protein glycation may interfere with the measurement of HbA1c. This includes inhibition of erythropoiesis or reduced RBC lifespan caused by uremic toxins. Impaired RBC turnover presents as anemia and is common in CKD, even in the early stages. Data from the National Health and Nutrition Examination Survey (NHANES) from 2007 to 2010 yielded an estimated prevalence of anemia ranging from 8.4% at stage 1 to 50.4% at stage 4 [39]. According to our previous study, we found that the association between HbA1c levels and outcomes existed in stage 3–4 CKD with Hb >10g/dL but not in stage 5 CKD [21,40]. Therefore, this cohort included patients with CKD stages 1–4 and excluded those with CKD stage 5 to decrease the possible effect of eGFR and ESA use on HbA1c readings. Although HbA1c levels are affected by a variety of CKD conditions, the alternative markers of glycated albumin and fructosamine are also influenced by hypoalbuminemia and have not been adequately validated in CKD populations [41,42,43]. Therefore, HbA1c remains the preferred glycemia biomarker despite its limitations.

Few studies have examined the predictive role of HbA1c in nondiabetic CKD. Trivin et al. presented a cohort of 1102 patients with CKD stages 1–4 in the absence of recognized diabetes and observed a higher risk of death in the high HbA1c tertile (5.7%–6.5%) [11]. No prior studies have stratified the HbA1c effect in association with MetS. However, it is noteworthy that the results of this study showed an association between elevated HbA1c levels and poor composite renal outcomes in nondiabetic CKD patients who concurrently had MetS. These findings suggest that for renal outcomes, the predictive role of HbA1c in the stage 1–4 nondiabetic CKD population depends on the presence or absence of MetS. As expected, individuals with metabolic abnormalities and high HbA1c levels had more unfavorable risk factors than metabolically healthy individuals that could slow CKD progression. Various biological mechanisms of kidney damage may be postulated, including MetS-related insulin resistance and glucose excursion of high HbA1c-related oxidative stress. Moreover, the results of this study indicated the correlation between HbA1c and the TyG index, which was proposed as an alternative marker for insulin resistance. In this context, HbA1c values might also be added to the risk factors, supplementary to MetS, for poor renal outcomes even in the nondiabetic CKD population.

Among patients in this study without MetS, HbA1c might be considered a nutritional reference with a better prognosis of renal outcomes. Several studies in the literature have already described that an HbA1c of <5.0% (reference 5.0 to <5.5% or 5.7%) increased the mortality risk with J-shaped relationships in the nondiabetic population [9,44,45]. Even in the nondiabetic CKD cohort by Trivin et al., a J-shaped relation between the HbA1c values and ESRD risk was also found in the univariate analysis [11]. Our results for renal outcomes without MetS are consistent with Trivin’s findings. The concept of a “reverse metabolism” has been raised for the paradoxical relationship between HbA1c and clinical outcomes, which indicates a negative impact on survival due to malnutrition or chronic disorders [46]. Similarly, in the CKD population, malnutrition–inflammation-induced protein energy wasting is a recognized factor for CKD progression [24]. The clinical implication may be that a lower HbA1c level is an indicator of malnutrition and especially plays an important role in patients without MetS. The inverse association between HbA1c and composite renal outcome in non-MetS deserves additional studies to elucidate possible nonglycemic determinants.

This study had the following important limitations: It relied on a single HbA1c level and MetS status measurement at baseline. Changes in HbA1c levels and MetS status may have been affected by coexisting medical conditions including kidney function progression. Furthermore, HbA1c might underestimate dysglycemia in the CKD condition. However, this cohort included patients with CKD stages 1–4 and excluded those with CKD stage 5 because the predictivity of HbA1c might be influenced most in advanced CKD [21]. Until now, HbA1c remains the preferred biomarker [42]. Additionally, our analysis resulted from a longitudinal observation, and this result could not clarify the interrelationship between HbA1c and MetS. Finally, since it was an observational study, it could not eliminate possible residual confounding by unmeasured risk factors.

## 5. Conclusions

In conclusion, high HbA1c levels are associated with poor renal composite outcomes in patients with nondiabetic CKD stages 1–4 with MetS. The prognostic value of HbA1c is modified by the presence of MetS and showed better outcomes in those without MetS, whereas the underlying mechanisms remain inconclusive. Whether malnutrition–inflammation could explain this modification deserves further study.

## Figures and Tables

**Table 1:** Baseline characteristics of non-diabetic CKD stage 1–4 patients.

Variable	Non-MetS				*p*	MetS				*p*
	All	HbA1c < 5%	HbA1c 5–5.7%	HbA1c ≥ 5.7%		All	HbA1c < 5%	HbA1c 5–5.7%	HbA1c ≥ 5.7%	
**No. of patients**	628	93 (14.8%)	347 (55.3%)	188 (29.9%)	*-*	642	54 (8.4%)	282 (43.9%)	306 (47.7%)	*-*
**Demographics**										
Age (year)	58.0 (17.3)	51.2 (21.0)	57.5 (16.8)	62.2 (15.1)	<0.001	62.8 (14.2)	59.5 (16.4)	63.2 (15.1)	63.0 (13.0)	0.205
Sex (female)	202 (32.2%)	36 (38.7%)	112 (32.3%)	54 (28.7%)	0.241	231 (36.0%)	19 (35.2%)	101 (35.8%)	111 (36.3%)	0.985
Cardiovascular disease	84 (13.4%)	11 (11.8%)	39 (11.2%)	34 (18.1%)	0.076	126 (19.6%)	9 (16.7%)	62 (22.0%)	55 (18.0%)	0.402
Hypertension	263 (41.9%)	36 (38.7%)	159 (45.8%)	68 (36.2%)	0.077	405 (63.1%)	39 (72.2%)	189 (67.0%)	177 (57.8%)	0.024
Hyperuricemia	106 (16.9%)	19 (20.4%)	54 (15.6%)	33 (17.6%)	0.515	165 (25.7%)	16 (29.6%)	72 (25.5%)	77 (25.2%)	0.784
Cancer	53 (8.4%)	9 (9.7%)	26 (7.5%)	18 (9.6%)	0.638	34 (5.3%)	2 (3.7%)	15 (5.3%)	17 (5.6%)	0.855
Malnutrition–inflammation ^1^	129 (20.5%)	29 (31.2%)	58 (16.7%)	42 (22.3%)	0.003	81 (12.6%)	15 (27.8%)	37 (13.1%)	29 (9.5%)	0.005
**Laboratory data**										
eGFR (ml/min/1.73 m^2^)	40.1 (26.5–56.2)	37.1 (25.0–65.3)	40.2 (26.3–56.2)	40.3 (29.9–51.8)	0.638	35.1 (24.5–47.9)	32.2 (20.1–44.5)	33.1 (22.8–46.7)	37.5 (27.1–49.7)	0.337
UPCR (mg/g)	451 (162–1171)	551 (199–1454)	436 (157–1051)	436 (147–1202)	0.310	608 (218–1368)	730 (260–1665)	578 (249–1350)	615 (203–1352)	0.208
Hemoglobin (g/dL)	12.4 (2.1)	12.2 (2.2)	12.5 (2.1)	12.4 (2.1)	0.440	12.6 (2.2)	11.4 (2.2)	12.6 (2.2)	12.9 (2.2)	<0.001
Albumin (g/dL)	4.0 (0.5)	3.9 (0.7)	4.0 (0.5)	4.0 (0.5)	0.112	4.0 (0.5)	3.9 (0.6)	4.0 (0.5)	4.1 (0.4)	0.037
ALT (mg/dL)	23.6 (17.2)	23.0 (16.2)	22.8 (15.1)	25.4 (21.0)	0.228	26.7 (23.3)	28.9 (23.8)	24.2 (27.1)	28.7 (18.9)	0.051
CRP (mg/L)	0.7 (0.2–2.8)	0.5 (0.1–2.8)	0.7 (0.3–2.2)	1.0 (0.2–4.7)	0.273	1.0 (0.4–4.0)	1.2 (0.5–5.3)	1.0 (0.4–3.3)	1.1 (0.4–4.4)	0.139
Phosphorus (mg/dL)	3.7 (0.8)	3.9 (1.1)	3.7 (0.7)	3.7 (0.7)	0.012	3.8 (0.8)	4.0 (1.0)	3.8 (0.8)	3.8 (0.7)	0.104
Calcium (mg/dL)	9.2 (0.6)	9.2 (0.6)	9.2 (0.6)	9.2 (0.6)	0.623	9.2 (0.7)	9.1 (0.8)	9.1 (0.7)	9.3 (0.6)	0.001
**Outcomes**										
Renal outcome ^2^	141 (22.5%)	32 (34.4%)	84 (24.2%)	25 (13.3%)	<0.001	182 (28.3%)	16 (29.6%)	90 (31.9%)	76 (24.8%)	0.318
All-cause mortality	78 (12.4%)	12 (12.9%)	37 (10.7%)	29 (15.4%)	0.277	110 (17.1%)	11 (20.4%)	50 (17.7%)	49 (16.0%)	0.691

Abbreviations: HbA1c: glycated hemoglobin; MetS: metabolic syndrome; eGFR: estimated glomerular filtration rate; UPCR: urine protein-to-creatinine ratio; ALT: alanine aminotransferase; ESRD: end-stage renal disease; CRP: c-reactive protein. Data are presented as mean (standard error), median (interquartile range), or count (percentage%). ^1^ Malnutrition–inflammation: MIS ≥ 8. ^2^ Renal outcome: end-stage renal disease +50% decline of eGFR.

**Table 2:** Baseline characteristics of components of metabolic syndrome in CKD stage 1–4 patients.

Variable	Non-MetS				*p*	MetS				*p*
	All	HbA1c < 5%	HbA1c 5–5.7%	HbA1c ≥ 5.7%		All	HbA1c < 5%	HbA1c 5–5.7%	HbA1c ≥ 5.7%	
**Components of metabolic syndrome**										
MetS scores	1.5 (0.7)	1.3 (0.8)	1.5 (0.7)	1.6 (0.6)	0.002	3.6 (0.7)	3.6 (0.7)	3.4 (0.6)	3.7 (0.7)	<0.001
Waist criteria	133 (21.2%)	14 15.1%)	73 (21.0%)	46 (24.5%)	0.191	500 (77.9%)	44 (81.5%)	201 (71.3%)	255 (83.3%)	0.002
Blood pressure criteria	425 (67.7%)	54 (58.1%)	245 (70.6%)	126 (67.0%)	0.070	589 (91.7%)	50 (92.6%)	260 (92.2%)	279 (91.2%)	0.879
HDL criteria	167 (26.6%)	28 (30.1%)	89 (25.6%)	50 (26.6%)	0.688	489 (76.2%)	43 (79.6%)	228 (80.9%)	218 (71.2%)	0.020
Blood sugar criteria	130 (20.7%)	13 (14.0%)	68 (19.6%)	49 (26.1%)	0.047	375 (58.4%)	28 (51.9%)	138 (48.9%)	209 (68.3%)	<0.001
Triglyceride criteria	69 (11.0%)	11 (11.8%)	31 (8.9%)	27 (14.4%)	0.153	342 (53.3%)	28 (51.9%)	144 (51.1%)	170 (55.6%)	0.539
**Associated data**										
Waist (cm)	80.8 (11.1)	77.1 (11.3)	81.3 (10.7)	81.7 (11.4)	0.002	92.5 (10.9)	93.9 (10.9)	90.3 (10.2)	94.4 (11.2)	<0.001
BMI (kg/m^2^)	23.1 (3.3)	22.4 (3.7)	23.2 (3.2)	23.3 (3.2)	0.093	26.4 (4.0)	25.7 (3.7)	25.7 (3.9)	27.2 (4.0)	<0.001
Systolic BP (mmHg)	131.1 (18.9)	129.1 (21.1)	130.9 (18.3)	132.4 (18.7)	0.377	138.6 (18.2)	139.9 (18.6)	137.9 (18.2)	139.1 (18.2)	0.630
Diastolic BP (mmHg)	79.3 (12.6)	78.1 (14.2)	79.9 (12.5)	78.7 (12.0)	0.345	82.5 (12.5)	83.2 (12.0)	82.6 (13.6)	82.2 11.6)	0.830
Total cholesterol (mg/dL)	194 (168–222)	187 (157–217)	199 (168–228)	191 (170–219)	0.108	192 (168–223)	188 (157–221)	188 (166–222)	196 (171–224)	0.819
Triglyceride (mg/dL)	98 (71–125)	95 (68–128)	93 (71–122)	102 (79–134)	0.692	155 (109–211)	152 (108–193)	150 (106–205)	162 (112–223)	0.919
HDL cholesterol (mg/d)	50.9 (15.2)	50.3 (16.6)	52.2 (15.8)	49.0 (13.0)	0.056	38.8 (11.1)	37.3 (9.9)	38.1 (11.2)	39.8 (11.0)	0.104
Blood glucose (mg/dL)	94.7 (16.0)	90.3 (13.1)	93.8 (10.7)	98.6 (23.2)	<0.001	103.8 (18.7)	101.7 (16.1)	98.9 (11.8)	108.6 (22.7)	<0.001
HbA1c (%)	5.5 (0.6)	4.6 (0.4)	5.4 (0.2)	6.1 (0.3)	<0.001	5.7 (0.6)	4.5 (0.4)	5.5 (0.2)	6.2 (0.4)	<0.001
TyG index	8.4 (0.5)	8.3 (0.5)	8.4 (0.5)	8.5 (0.5)	0.003	9.0 (0.5)	8.9 (0.7)	9.0 (0.5)	9.1 (0.5)	<0.001

Abbreviations: HbA1c: glycated hemoglobin; MetS: metabolic syndrome; HDL: high density lipoprotein cholesterol; BMI: body mass index; BP: blood pressure; TyG index: triglyceride-glucose index. Data are presented as mean (standard error), median (interquartile range), or count (percentage%).

**Table 3:** Linear regression for HbA1c.

Variables	Beta Coefficient (95% CI)	*p* Value
Age (years)	0.004 (0.002 to 0.007)	<0.001
Sex (female vs. male)	0.034 (−0.040 to 0.108)	0.362
Cardiovascular disease	0.068 (−0.024 to 0.159)	0.146
Smoking	0.074 (−0.024 to 0.172)	0.137
**MetS Components**		
Waist criteria (+)	0.114 (0.049 to 0.179)	0.001
Blood pressure criteria (+)	0.092 (0.000 to 0.184)	0.050
HDL criteria (+)	−0.056 (−0.122 to−0.009)	0.091
Blood sugar criteria (+)	0.158 (0.090 to 0.225)	<0.001
Triglyceride criteria (+)	−0.024 (−0.122 to 0.075)	0.637
**Laboratory data**		
eGFR (mL/min/1.73 m^2^)	0.000 (−0.002 to 0.001)	0.889
Log-UPCR	0.023 (−0.041 to 0.088)	0.447
Hemoglobin (g/dL)	0.023 (0.004 to 0.042)	0.019
Albumin (g/dL)	0.007 (−0.071 to 0.085)	0.863
Log-CRP	0.040 (0.002 to 0.078)	0.038
Phosphorus (mg/dL)	−0.030 (−0.074 to 0.015)	0.188
Malnutrition–inflammation *	−0.056 (−0.114 to −0.001)	0.043
TyG index	0.165 (0.080 to 0.250)	<0.001

Abbreviations: HbA1c: glycated hemoglobin; MetS: metabolic syndrome; eGFR: estimated glomerular filtration rate; UPCR: urine protein-to-creatinine ratio; HDL: high density lipoprotein cholesterol; TyG index: triglyceride-glucose index. * Malnutrition–inflammation was defined as malnutrition–inflammation score ≥ 6.

**Table 4:** Association between HbA1c and clinical outcome with or without metabolic syndrome.

	Non-MetS			MetS		
	Hba1c < 5%	Hba1c 5–5.7%	Hba1c ≥ 5.7%	Hba1c < 5%	Hba1c 5–5.7%	Hba1c ≥ 5.7%
**HR for renal outcome**						
Unadjusted	1 (reference)	0.57 (0.38–0.85) *	0.30 (0.18–0.50) *	1 (reference)	0.87 (0.51–1.49)	0.64 (0.37–1.10)
Fully adjusted	1 (reference)	0.49 (0.32–0.77) *	0.25 (0.14–0.45) *	1 (reference)	2.04 (1.11–3.74) *	2.00 (1.06–3.78) *
**HR for all-cause mortality**						
Unadjusted	1 (reference)	0.78 (0.40–1.49)	1.14 (0.58–2.24)	1 (reference)	0.76 (0.40–1.46)	0.67 (0.35–1.29)
Fully adjusted	1 (reference)	0.65 (0.34–1.24)	0.57 (0.29–1.11)	1 (reference)	0.93 (0.47–1.82)	1.00 (0.51–1.98)

Fully adjusted model: adjusted for age, sex, eGFR, log UPCR, cardiovascular disease, cancer, severe liver disease, smoker, HTN, malnutrition–inflammation, Hb, albumin, log CRP, phosphorus, BMI, waist, mean BP, HDL cholesterol, and log TG. * *p* value < 0.05.

## Data Availability

Not applicable.

## Supplementary Materials

The following supporting information can be downloaded at: https://www.mdpi.com/article/10.3390/biomedicines10081858/s1, Table S1: Logistic regression for HbA1c; Table S2: Association between metabolic syndrome and clinical outcomes.
