# Supplementary material for: Predictive Value of HbA1c and Metabolic Syndrome for Renal Outcome in Non-Diabetic CKD Stage 1–4 Patients
Source: Biomedicines. 2022 Aug 2;10(8):1858. doi: 10.3390/biomedicines10081858 (PMC9404918; doi:10.3390/biomedicines10081858)
Supplement: Supplementary file 1 [file biomedicines-10-01858-s001.zip › biomedicines-1765947-supplementary.pdf]

Supplement Table S1. Logistic regression for HbA1c

Variables	Odds ratio (95% CI)	P value
Age (years)	1.014 (1.005 to 1.024)	0.002
Sex (female vs male)	1.081 (0.809 to 1.445)	0.598
eGFR (ml/min/1.73 m^2)	1.001 (0.996 to 1.007)	0.627
Log-UPCR	1.105 (0.860 to 1.420)	0.434
Cardiovascular disease	1.067 (0.758 to 1.502)	0.711
Smoking	1.131 (0.776 to 1.649)	0.523
Hemoglobin (g/dl)	1.052 (0.977 to 1.132)	0.179
MetS componenets		
Waist criteria (+)	1.635 (1.275 to 2.095)	<0.001
Blood pressure criteria (+)	1.033 (0.755 to 1.412)	0.840
HDL criteria (+)	0.901 (0.702 to 1.156)	0.413
Blood sugar criteria (+)	2.074 (1.628 to 2.642)	<0.001
Triglyceride criteria (+)	1.596 (1.224 to 2.082)	0.001
Malnutrition-inflammation*	0.801 (0.642 to 0.953)	0.038
Albumin (g/dl)	0.912 (0.670 to 1.242)	0.558
Log-CRP	1.279 (1.100 to 1.487)	0.001
Phosphorus (mg/dl)	0.936 (0.787 to 1.114)	0.457

Abbreviations: HbA1c: glycated hemoglobin; MetS: metabolic syndrome; eGFR: estimated glomerular filtration rate; UPCR: urine proteinr-to-creatinine ratio; HDL: high density lipoprotein cholesterol;

* Malnutrition-inflammation was defined as malnutrition-inflammation score ≥6.

Supplement Table S2. Association between metabolic syndrome and clinical outcomes.

	IDF definition as Table 1-4	
	**Non-METS**	**METS**
**HR for renal outcome**		
Unadjusted	1 (reference)	1.35 (1.08-1.68)*
Fully-adjusted	1 (reference)	1.31 (1.01-1.69)*
**HR for all-cause mortality**		
Unadjusted	1 (reference)	1.33 (1.00-1.77)*
Fully-adjusted	1 (reference)	1.27 (0.92-1.74)

Fully-adjusted model: adjusted for age, sex, eGFR, log UPCR, cardiovascular disease, cancer, severe liver disease, smoker, HTN, malnutrition-inflammation, Hb, albumin, log CRP, phosphorus, BMI, waist, mean BP, HDL cholesterol, and log TG. * p value <0.05.
